# A Novel Approach to the Identification of Compromised Pulmonary Systems in Smokers by Exploiting Tidal Breathing Patterns

**DOI:** 10.3390/s18051322

**Published:** 2018-04-25

**Authors:** Raj Rakshit, Anwesha Khasnobish, Arijit Chowdhury, Arijit Sinharay, Arpan Pal, Tapas Chakravarty

**Affiliations:** TCS Research and Innovation, Kolkata-700156, India; arijit.chowdhury2@tcs.com (A.C.); arpan.pal@tcs.com (A.P.); tapas.chakravarty@tcs.com (T.C.)

**Keywords:** tidal breathing pattern, pulmonary ailments, locally weighted learning, ridge regression

## Abstract

Smoking causes unalterable physiological abnormalities in the pulmonary system. This is emerging as a serious threat worldwide. Unlike spirometry, tidal breathing does not require subjects to undergo forceful breathing maneuvers and is progressing as a new direction towards pulmonary health assessment. The aim of the paper is to evaluate whether tidal breathing signatures can indicate deteriorating adult lung condition in an otherwise healthy person. If successful, such a system can be used as a pre-screening tool for all people before some of them need to undergo a thorough clinical checkup. This work presents a novel systematic approach to identify compromised pulmonary systems in smokers from acquired tidal breathing patterns. Tidal breathing patterns are acquired during restful breathing of adult participants. Thereafter, physiological attributes are extracted from the acquired tidal breathing signals. Finally, a unique classification approach of locally weighted learning with ridge regression (LWL-ridge) is implemented, which handles the subjective variations in tidal breathing data without performing feature normalization. The LWL-ridge classifier recognized compromised pulmonary systems in smokers with an average classification accuracy of 86.17% along with a sensitivity of 80% and a specificity of 92%. The implemented approach outperformed other variants of LWL as well as other standard classifiers and generated comparable results when applied on an external cohort. This end-to-end automated system is suitable for pre-screening people routinely for early detection of lung ailments as a preventive measure in an infrastructure-agnostic way.

## 1. Introduction

Pulmonary diseases are evolving to be one of the major life-threatening ailments throughout the world in recent times [1]. Both urban as well as rural populations are affected by the devastating effects of pulmonary ailments such as asthma, pneumothorax, obstructive airway diseases (OAD), chronic obstructive pulmonary disease (COPD), apnea, and pulmonary fibrosis. These are caused by pathophysiological conditions and are often accelerated by smoking, pollution, and environmental effects. According to the World Health Organization (WHO), the primary cause of COPD is exposure to tobacco smoking (either active or passive smoking) [2]. Globally, it is estimated that about 3 million deaths were caused by COPD in 2015 (i.e., 5% of all deaths globally in that year) [3].

Unfortunately, most of the pulmonary diseases are irreversible. They can obviously be treated, the efficacy of which is predominantly dependent upon the stage of its detection. To hinder the progression of a chronic disease, early detection and management of the disease via routine screening is hence crucial. Many times pulmonary diseases are misdiagnosed; for instance, COPD is often diagnosed as asthma and treated as such. The consequences of these delays and/or misdiagnoses adds more to the patients’ woes and can also prove to be fatal.

The most common and prevalent way to perform an adult pulmonary function test (PFT) for the diagnosis of pulmonary conditions is Spirometry [4]. Due to the requirement that patients cooperate, spirometry can only be performed on children (above 6 years of age) and patients who are able to understand and follow instructions. Moreover, because of the effort needed, Spirometry cannot be performed when patients have hemoptysis of unknown origin, pneumothorax, an unstable cardiovascular status, thoracic, abdominal, or cerebral aneurysms, cataracts, or have had recent eye, thoracic, or abdominal surgery.

Lately, in order to eliminate the need for the forceful breathing maneuvers of spirometry, researchers have started investigating a new line of pulmonary evaluation through tidal breathing analysis (TBA). TBA is basically acquiring restful breathing parameters and analyzing tidal expirograms (tidal breathing flow rate (TBF(t)) or flow versus volume, also known as Tidal breathing flow-volume (TBFV) loops). From its inception in the early 1950s as a mere ventilation tool for anesthetized patients undergoing surgeries, TBA became the stand-alone modality for the infant pulmonary function test (PFT) in the following four decades. Recently, a plethora of research was conducted incorporating adult TBA to find pathological signatures of pulmonary ailments. In our previous work [5,6], we demonstrated a tidal breathing pattern recorder (TBPR), a novel phase-sensing-based equitable ultrasound sensor platform capable of recording and computing adult tidal breathing parameters.

The aim of this paper is to evaluate whether tidal breathing signatures can indicate a deteriorating adult lung condition in an otherwise healthy person. If successful, such a system can be used as a pre-screening tool for all people before some of them need to undergo a thorough clinical checkup. It is a well-known fact that smoking habits degrade lung condition [7,8,9] and therefore we tested our tidal-breathing-based approach to see if we can detect or discriminate between smokers’ compromised lungs and non-smokers’ lungs. Thus, our objective resides on evaluating “tidal breathing” only as a marker for lung condition. We have considered a group of participants (smokers and non-smokers) who do not have any known history of pulmonary ailments and are otherwise healthy. Tidal breathing patterns are acquired during restful, effortless breathing of adult participants. Thereafter, physiological attributes are extracted from the acquired tidal breathing signals. An intelligent machine learning technique is implemented to classify smokers and non-smokers from the extracted tidal breathing features.

One of the crucial parameters of TBA is tidal volume, whose variation needs to be handled for proper analysis. The tidal volume rides on top of functional residual capacity (FRC), which is normally assumed to be 2.4–3 L for healthy subjects [10], but varies widely with age [11], demography [12], and even with anthropometric determinants [13] within the same ethnic and age groups. Deviations in FRC lead to wide baseline variation in acquired tidal breathing expirograms. The standard approach to deal with such varying patterns is to implement feature normalization prior to classifier training. However, each time a new instance (feature set from a new subject) is introduced to the classifier, if it lies outside the range of the training set then normalization needs to be repeated and the classifier retrained. This increases the cost of computation and may not be desirable for implementation in real-time. The primary novelty of this paper lies in envisioning a machine learning approach which mitigates the need for normalizing feature values. This unique approach first finds an appropriate neighborhood for the subject under consideration in which all other instances will have same baseline (lung capacity). The algorithm finally arrives at an intelligent decision regarding the placement of the subject across either side of the decision boundary. This ensures that each subject is compared against instances with the same lung capacities in the vicinity and no global normalization of features is required, neither in the training nor in the testing phase.

We do not aim to replace spirometry and/or other clinically graded pulmonary diagnostic tests. Rather, we propose the developed system (comprising the tidal breathing data acquisition and novel data driven analysis technique) as a user-friendly and infrastructure-agnostic early pulmonary pre-screening tool. In order to hinder the progression of a chronic disease, early detection and management of the disease via routine screening are very crucial. The pulmonary function tests are done at an advanced stage of disease progression and are generally taken by patients upon a doctor’s prescription. In contrast, our system is mainly aimed towards generating mass awareness via daily routine check-ups which might give an indication towards further medical attention.

The rest of the paper is organized as follows. Section 2 provides an overview on related work and is followed by a description of the methodology implemented in this work in Section 3. The results of TBA are discussed in detail in Section 4. Finally, we conclude and discuss future work in Section 5.

## 2. Related Work

We start our exploration by articulating a short background on the physiology of tidal breathing for both infant and adult lungs and different modalities to acquire tidal breathing data.

### 2.1. TBA: A Physiological Modality of Pulmonary Artifact Detection

TBA gained interest as a physiological technique to monitor/diagnose/screen pulmonary artifacts in the early 1950s [14]. Thorough research incorporating TBA on adults [15,16,17,18], children/infants [19], and lung-models [20] led to newer and safer ventilation guidelines. Morris et al. [21], in 1981, first reported the potential of TBF(t) to detect pulmonary abnormalities, which paved the way for TBA to become a powerful alternative to traditional spirometry. With further insight, TBA became the “stand-alone” modality of airflow obstruction measurement [22] for children and infants in the mid-1990s.

Following the contemporary global interest in TBA, in 2000, the European Respiratory Society (ERS)/American Thoracic Society (ATS) Task Force came up with international standards and recommendations for TBA [23,24,25]. They pinpointed the need and guidelines for some of the practical issues with TBA, such as, (a) Automatic segmentation of breaths into expiration and inspiration phases, (b) Detection of onset of inspiration and expiration in acquired TBF(t), and (c) Derivation of drift-free TBV(t) from TBF(t).

In the following years, focus shifted from analyzing a single feature at a time to detailed structural [26,27], temporal [28], and spectral analysis [29] of the recorded tidal expirograms. Automated detection of pulmonary artifacts was also attempted on several occasions with varying degrees of success [30,31]. In 2007, ATS and ERS officially approved TBA as a modality of PFT for pre-school children [32].

### 2.2. TBA of Compromised Adult Lungs: The Paradigm Shift

From the very beginning of the 21st century, TBA of adults slowly started drawing the attention of researchers across the globe. Researchers investigated the Expiratory Flow Limitation during Tidal Breathing (EFLT), which occurs when expiratory flow does not increase in accordance with the trans-pulmonary pressure in patients with pulmonary artifacts. EFLT causes inhomogeneity in ventilation distribution with simultaneous compromised gas exchange. Moreover, uneven distribution of stress and strain within the tracheal tree causes small airway injury to overt COPD in smokers [33,34]. With the establishment of the fact that tidal expirograms can be used to identify and quantify the stage of airflow disease, several interesting research outcomes have manifested in recent years. Ma et al. [16] performed a breath-by-breath analysis of a tidal breathing signal and came up with quantifications of TBFV curve concavity. They described progressive shape changes denoting EFLT during incremental exercise in COPD patients. Williams et al. [15] derived and compared the centroid coordinates (geometric center) of tidal expirograms of healthy and COPD lungs and showed that the centroids shift to the left with increasing airflow obstruction. Chiari et al. [35] analyzed the tidal breathing of COPD patients and showed a correlation of EFLT with COPD. TBA over the years has proven to be a powerful tool to assess respiratory systems.

### 2.3. Tidal Breathing Data Acquisition Techniques

Tidal breathing signals are recorded either directly or indirectly. In direct measurements, accurate breath-by-breath tidal expirograms are obtained directly by placing sensors at or near the airway openings. A pneumotachgram (PNT) is a simple device which acquires respiratory flow signals by relying on the Venturi effect [36]. Unfortunately, it affects the very parameter it was devised to measure by inducing dead space and elevating CO_2_ levels, which alter the tidal breathing pattern [37]. Thus, despite its simplicity, this particular modality of measurement was questioned severely. In the ERS/ATS international guidelines for TBA [23], the use of the dead-space-free-flow-through technique was recommended and was supported by subsequent research [38,39]. The first prototype system for the direct measurement of tidal breathing using ultrasonic flow meters (UFM) was demonstrated in 2001 [40]. It complied with the ERS/ATS guidelines. To the best of the authors’ knowledge, all of these prototypes and commercial UFMs inherently use the pulsed diagonal-beam and transit-time tracking based technique, which was first reported in 1986 by Buess et al. [41].

Indirect measurements, on the other hand, monitor spatio-temporal variations of other tissues associated with breathing, such as chest wall, abdominal wall, and esophageal pressures. These are suitable for longitudinal observation of respiration patterns or tracking the temporal disparity between thoracic and abdominal movements. The most common method of indirect measurement is respiratory inductance plethysmography (RIP), which was first demonstrated by Konno et al. [42]. In RIP, a subject’s respiratory movements cause a change in the inductance created by fine tungsten wires inside a compliant elastic belt wrapped around the subject. This in turn alters the resonant frequency of the overall circuit that is being tracked. Fibre-optic respiratory plethysmography [43] is principally the same as RIP, where instead of tungsten wires fiber-optic loops are used, which alters its radius of curvature and hence causes variation in the beam of light passing through it in accordance with a subject’s chest wall movement with respiration. Electrical Impedance tomography [44] provides measurements of spatial distribution of tidal breathing for both adults [45] and infants [46].

## 3. Methodology

**Figure 1:**
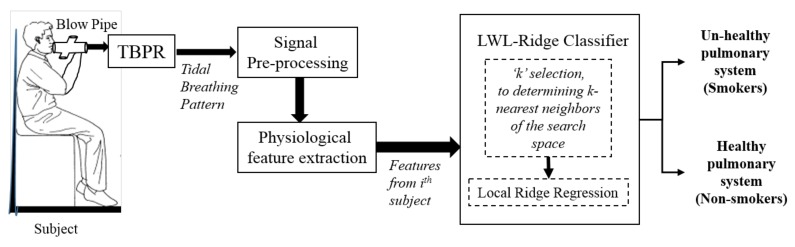
Schematic of the overall system for healthy and unhealthy pulmonary system recognition from tidal breathing signals. TBPR: tidal breathing pattern recorder; LWL-Ridge: locally weighted learning classifier with ridge estimator.

This section presents the complete sensor architecture, which comprises a three-dimensional (3D) printed blow pipe (fitted with ultrasound Tx and Rx), phase detection electronics, and a personal computer (PC)/Laptop running data acquisition and visualization graphical user interface (GUI) software as illustrated in Figure 1. Each component is described in the following sub-sections.

### 3.1. Overall System

The overall system architecture is illustrated in Figure 1. Our approach requires the subject to breathe effortlessly through the blow pipe of the TBPR for data acquisition. The acquired signal is preprocessed to remove the artifacts, extract the breath-by-breath signals, and to derive the tidal volumetric signals. Physiological parameters are extracted from the flow and volumetric tidal breath signals. These physiological parameters are then fed to the locally weighted learning classifier with ridge estimator (LWL-Ridge), which then discriminates between smokers’ unhealthy and non-smokers’ healthy lung conditions.

### 3.2. Tidal Breathing Pattern Acquisition

In our previous works [5,6], we exhibited the complete mechanism of the ultrasonic phase-measurement-based sensor platform, TBPR, suitable for the acquisition of tidal breathing patterns from non-sedated adults. The system was calibrated against a medically graded 3 L syringe. Upon validation with a commercial spirometer, the maximum error was found to be 16.25% (where the mean error was 12.04% with a standard deviation of 4.70%) [6]. TBPR records TBF(t) for the cascaded data analytics stage and the volumetric information is suitably derived from the same. The developed TBPR acquires tidal breathing signals from the phase shift between the transmitted and received ultrasound when breathing is injected, unlike transit-time tracking in contemporary UFMs. This offers a simple electronics design and better sensitivity. We have tried our best to comply with ERS/ATS recommendations related to flow-through hardware design for adult subjects. The proposed system incorporated an absolutely hollow blow-pipe, an end-to-end flow-through design, a small apparatus length, and orthogonal hand-held positioning (with respect to the subject’s mouth).

### 3.3. Experimental Setup

Twenty volunteers (6 females and 14 males, 10 smokers and 10 nonsmokers), denoted as “Cohort-1”, with no history of major pulmonary artifacts (inferred from the Tata Consultancy Services (TCS) annual health check-up program) and a moderately active lifestyle from our lab took part in the tidal breathing data acquisition using TBPR. All of the experimental procedures are Helsinki declaration [47]-compliant and ethical clearance was sought from the Tata Consultancy Services (TCS) institutional review board (Ref. no. TCS_IRB/ESD_TBPR, dated 27 April 2017). Table 1 reveals the demographics of the participants; the tabular format is adopted from [48]. The subjects of “Cohort-2” participated in the next experimental phase in order to evaluate the performance of the devised system (explained in Section 4.6).

The subjects were made to sit comfortably with their arms and back at rest. The objective and procedures of the experiment were explained to them. Each subject held the blow-pipe in their palm orthogonally with their mouth and kept their arms at rest. They were instructed to blow in and out spontaneously without any additional effort through the blow pipe for 60 s. Air passage via nostrils was blocked using a nose clip. Three such trials, one trial each day for three consecutive days, were conducted for each subject. It was found that with sessions of more than one minute in duration, the breathing patterns start changing due to human factors. In fact, when asked, the subjects complained about their discomfort while keeping the blow pipe inside their mouth for such extended durations even for performing restful breathing. Although the time limit after they started complaining varied across subjects, we kept the session length at one minute and conducted three such sessions with each participant to ensure the subjects’ collective co-operation. We used an Atmega32 microprocessor and the python interface to record the tidal breathing data at a sampling rate of 100 Hz. The experimenter gave visual cues to the participants about starting and stopping breathing through the pipe and observed the real-time breathing signals during the entire 60 s of experimentation. Subjects were instructed to redo the experiment if any artifacts, such as a sudden sneeze, cough, and/or movements occurred. The experimenter’s screen, which had the real-time breathing signal streaming on it, was faced away from participants to negate unwanted interference via visual feedback.

### 3.4. Signal Preprocessing and Feature Extraction

**Figure 2:**
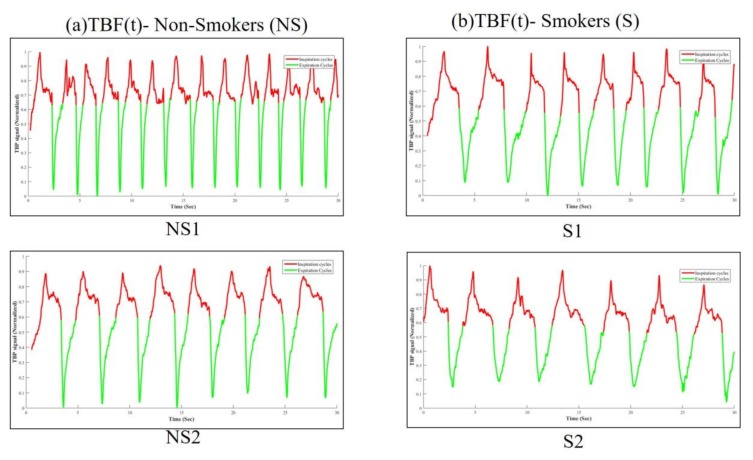
Preprocessed tidal breathing flow rate (TBF(t)) signals of (**a**) two non-smokers (NS1, NS2) and (**b**) two smokers (S1, S2). The red portions of the signal indicate inspiration and the green portion indicates expiration.

We followed the signal processing guidelines prescribed by the ERS/ATS Task force [23] to compute drift-free temporal and volumetric tidal breathing signals from the acquired tidal breathing data. The complete mathematical description of computing the tidal breathing waveforms from the calibrated sensor is outlined in our previous work [6]. This covers the criteria for automatic breathing cycle detection and locating onset/offsets of inspiration/expiration phases. Figure 2 shows respiratory traces, here TBF(t), of two non-smokers (in column 1) and two smokers (in column 2). The TBF(t) signals shown in Figure 2 were obtained after preprocessing (these steps are explained in detail in [6]) of the raw signal. We further computed several physiological attributes from the tidal breathing signals, which were found in tandem with the values reported in the State-of-the-Art studies [15,16,17]. In this present work, we are considering those computed physiological attributes as features to be fed to the classifier. Table 2 briefly summarizes the extracted features.

The inspiratory time (TI) and the expiratory time (TE) are highly affected during any obstructive airway disorder. People suffering from various pulmonary disorders tend to have altered peak flow values. Smoking habits, gender, and demography have an effect on the peak flow values. In the case of normal restful tidal breathing, the volume of air inspired is almost the same as the volume of air expired.

### 3.5. Classification Model

One of the main foci of this present work is to model a classifier algorithm which can deal with the variations in the tidal breathing pattern arising due to subjective variations of FRC. We used the locally weighted learning (LWL) model [49], which implements logistic regression internally. The LWL selects *k*-nearest neighbors for every observation and fits a hyper-plane locally within the *k*-neighbors using the underlying logistic regression. As the neighbors of every instance (subject) will have a similar lung capacity, normalization of features is not required. Moreover, in every *k*-neighborhood of instances, the features are expected to be highly correlated locally as they belong to a similar lung capacity neighbourhood. Thus, we used logistic regression with ridge estimators, also called Ridge-regression [50], which works well in the presence of multi-collinearity among the features [51].

Suppose we have a binary variable *S* (smoker or nonsmoker) and want to model its dependency on a vector ***x***, one vector for each trial of each subject, (here 60 such vectors). Each vector (denoted by *S_F_*) has length *p* (no of features), where *p* denotes the number of explanatory variables as given by Equation (1).

(1) $$\begin{array}{l} E(S)=1\;\cdot \;P(S=1)+0\;\cdot \;P(S=0)\\ =P(S=1)\\ =g(\beta^{\prime }\;S_{F}), \end{array}$$

where $\beta^{\prime }$ is a vector of length *p.* A common choice for *g*(*t*) is the inverse of the standard logistic distribution function given by Equation (2).

(2) g(t)=exp (t)/{ 1+exp (t) }

In this case, Equation (1) can be rewritten as Equation (3).

(3) $$logit\{P(S=1|x)\}=\beta^{\prime }\;x$$

where *logit*(*t*) *= log*{*t*/(1 *− t*)}. Equation (3) is a logistic regression model.

Ridge regression penalizes the magnitude of regression coefficients based on the L2 norm (Euclidian distance). The primary objective of Ridge regression is to minimize the Residual Sum of Squares (RSS) + *R* * sum of the square of coefficients, where *R* is called the ridge parameter, where R≥0. Setting *R* = 0 makes it ordinary linear regression. As *R* increases, regression coefficients reduce in magnitude.

If we have N observations, let Y denote the N × 1 matrix of all observations and let X be a matrix of dimension N × *p* whose rows correspond to the corresponding independent variable (features) for each observation. Then, the regression model can be described by Equation (4).

(4) $$Y=X\beta +\in_{N\times 1}$$

Here, $\in ={\left[\varepsilon_{i}\right]}_{N\times 1}$ and $\varepsilon_{i}$ is N(0,σ). The ridge regression parameters obtained for this model are given by Equation (5).

(5) $$\hat{\beta }={\hat{\beta }}_{ridge}={\left(X^{\prime }\;X+RI_{p}\right)}^{-1}\;X^{\prime }\;Y$$

For an ordinary least square (OLS) solution, $\beta ={\left(XX\right)}^{-1}\;X\;Y$. For *R* = 0, Equation (5) reduces to an ordinary least square solution with ${\hat{\beta }}_{ridge}={\left(X^{\prime }\;X+RI_{p}\right)}^{-1}\;X^{\prime }\;Y$. This is the Ridge estimator.

Ridge regression leads to a biased estimator but reduces variance. For Ridge regression, the mean squared error, *MSE = bias*^2^ *+ variance*, hence although bias is introduced, the overall MSE can decrease leading to better performance [50].

## 4. Results and Discussion

This section presents the results of the classifier model selection as well as the classification outputs. The statistical evaluation and comparisons with similar related works are described in the final sub-sections.

### 4.1. Feature Level Discriminability

**Figure 3:**
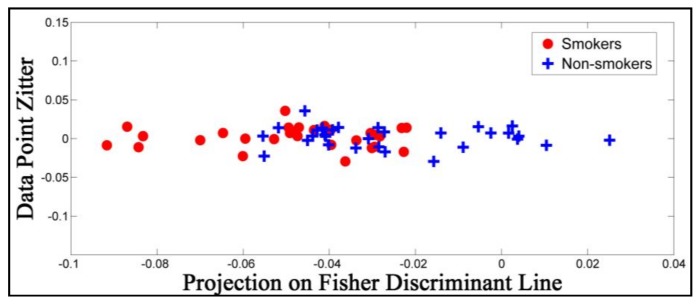
Projection of features on the Fisher’s Linear Discriminant Line.

The features that we have extracted are the physiological attributes computed directly from the acquired tidal breathing patterns. These primary physiological features and their linear or proportional combination have been used to classify compromised adult lungs. To inspect the effectiveness of these features, we have used the Fisher’s Linear Discriminant (FLD) method [52], which projects the computed 12-dimensional feature-set onto a single dimension. Figure 3 shows the features projected on the Fisher’s Linear Discriminant Line. The y-axis values are given a random and deliberate spread for visual aid and the x-axis presents the values of projection. This shows the formation of two clusters (red versus blue dots) with some overlaps. This indicates the usefulness of the computed tidal-breathing-based feature-set. Although FLD could be used for classification, it may lead to a weak classifier as is visible in Figure 3. In order to obtain higher accuracy and precision, we have employed a superior classifier as discussed in the following sub-sections.

### 4.2. Selection of Ridge Regression for LWL

The LWL-Ridge classifier is compared with three closely related variants of LWL classifiers for validation of our classification method selection. We investigated the performance of all of these classification schemes on the acquired dataset using fivefold cross validation. We used the open source WEKA [53] machine learning platform to run all of the classifiers with their default parameter values. Apart from the percentage accuracy (% Acc) of classification, several other well-known metrics of classifier evaluation [54,55] were investigated. These include True Positive Rate (TPR), True Negative Rate (TNR), F value, Kappa statistics, Area Under the Receiver Operating Characteristic Curve (AUC), and Area Under the Precision-Recall Curve (AUP). The best classifier is chosen among these based on the computed metrics. Table 3 lists the mean values of these metrics over 10 trials and their corresponding standard deviations are shown in parenthesis.

In Table 3, the best values obtained for each metric are highlighted. Our objective is to choose the best variant of the LWL classifier among these. LWL-ridge (LWL + L-R) conferred the best values for five metrics, viz., % Acc, TNR, Kappa value, AUC, and AUP. This indicates that LWL + L-R not only yields the highest classification accuracy of 85%, but also has highest specificity of 90%. The metrics of Table 3 support our selection of LWL-ridge (LWL + L-R) as the most appropriate among the other variants of LWL classifier combinations.

### 4.3. Determination of LWL-Ridge Parameters: K and R

The parameters of LWL + L-R (LWL-ridge) are tuned to attain the most effective classifier performance. The two parameters are the *k* value in the *k*-nearest neighborhood of LWL and the ridge parameter (*R*) value of ridge-regression. We varied *R* and *k*, and trained/tested the classifier on the randomly split dataset. The results are tabulated in Table 4.

**Figure 4:**
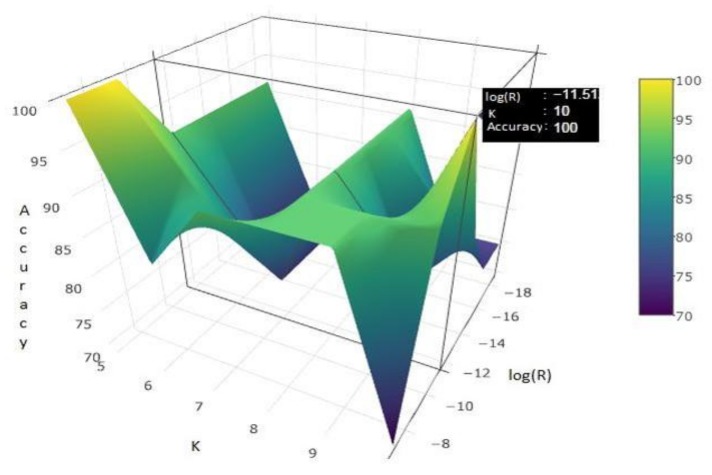
Surface plot for 80–20 split.

In Table 4, T denotes the number of test instances. We varied T as T = 1/*t* of the total instances (i.e., 60 × 1/*t*) and t∈{2, 3,…, 7}. This corresponds to *t* = number of test instances = 30, 20, 15, 12, 10, 9 for *t* = 2, 3, 4, 5, 6, 7, respectively. As a rule of thumb, *k* should be the square root of the number of instances in training [55]; hence, we varied *k* from 5 to 10 and varied R from 10^−3^ to 10^−8^. So, the parameter tuning involves all possible combinations of the triplet depicted in Equation (6).

(6) $$\left\{T,k,\;R\}=(1/t,k,10^{-r}\right)$$

where t∈{2,3,…,7}, k∈{5,6,…10} and r∈{3,4,…8}. Out of all of the possible cases, three cases attained 100% accuracy for the following choice of triplets:$\{T,\;k,\;R\}=\left\{\;1/5,\;10,\;10^{-5}\right\}\;,\;\left\{\;1/5,\;5,\;10^{-4}\right\}\;,\;\left\{\;1/5,\;5,\;10^{-3}\right\}$ as illustrated in Figure 4 and Table 4.

These three observations indicate that the classifier performs best when we take 1/5 of the total dataset as test cases (80% as training cases and 20% as test cases). To choose the optimal value of *k* and $\hat{\beta }$ out of these three choices, we investigated the accuracy (in percentage) around the 80–20 (1/5 of the entire data for the test) split. We discarded the 1st choice, namely $\{T,k,R\}=\left\{\;1/5,\;10,\;10^{-5}\right\}\;$, as it has maximum deviation in accuracy around the obtained maximum accuracy point for different splits. From the other two choices we found the optimum value of *k* to be 5.

To find the optimum value of *R* out of the possible two choices (i.e., 10^−3^ and 10^−4^), we ran a five-fold cross-validation (CV) and observed a few crucial metrics of classification. We chose five-fold because it matches with the 80–20 split (i.e., *T* = 1/5), at least with respect to the number of data in the test case. Table 4 lists the classification metrics after fivefold CV for the two choices of *R*. Table 5 lists the mean values of the metrics over all the trials, and their corresponding standard deviations are shown in parenthesis.

Comparing the performance metrics of untuned and tuned LWL with ridge-regression, we clearly observe that with appropriate tuning of the parameters, i.e., (*k*, *R*) = (5, 10^−3^), all of the classification performance metrics improve, except for a slight degradation in Kappa value. The final accuracy post-parameter tuning reaches 86.17%. To validate the robustness of the tuned classifier even further, we synthetically generated simulated datasets and fed them to the classifier, which is discussed in the following section.

### 4.4. Performance of Chosen Tuned Model on Simulated Dataset

To predict the accuracy of the classifier with an increased data size and/or to find the optimum training set dimension, we have synthetically generated instances from the recorded 60 instances and fed them to the classifier. The SMOTE [56] algorithm was used to generate these synthetic instances. It should be noted that SMOTE is generally used to take care of datasets with class imbalance, where it has been shown that oversampling the minority class along with undersampling the majority class performs better than only random majority undersampling. Here, we are using SMOTE to check the robustness of the tuned model parameters and to simulate probabilistic performance.

Table 6 illustrates classification performance with an increased dataset (actual data plus synthetically simulated data) based on the same metrics as are reported in Table 3. Each row of Table 6 summarizes one trial session with SMOTE. In every session out of the total number of instances, 60 are real data whereas the rest are generated using SMOTE, and the method of validation is fivefold CV repeated 10 times. As can be seen with only twice the number of instances, the accuracy is enhanced to 94% and with data sizes seven times higher, both the AUC and the AUP become 1.0. This is a marker of better performance with increased data size with the same set of features. This analysis will help us design our future trials and data-collection protocols.

### 4.5. Statistical Test

The performance of our classifier model, i.e., LWL-ridge (LWL + L-R), is validated against three standard classifiers, including support vector machine with radial basis function kernel (SVM-RBF) [57], random forest [58], and the *k*-nearest neighbor classifier (*k*-NN) [59], using the Friedman Statistical Test [60]. Table 7 outlines the classification metrics (mean values for all of the trials) for all of the above-mentioned classifiers. SVM-RBF and random forest were executed on the normalized feature set. They performed even worse when the feature set was not normalized. Since the performance of *k*-NN and (LWL + L-R) was exactly the same for both a normalized and non-normalized feature set, we have presented their result metrics for the non-normalized feature set. The *k* value of *k*-NN is set as 5 to maintain parity for comparison with our classifier, as the LWL-ridge uses *k* = 5. All of the classifiers perform equivalently according to the null hypothesis. Equation (7) presents the Friedman Statistic for *d* − 1degrees of freedom. The Friedman test for the null hypothesis follows a chi-square distribution ($\chi_{F}^{2}$) with (*d* − 1) degrees of freedom [60].

(7) $$\chi_{F}^{2}=\frac{12N}{d(d+1)}\left[\sum_{j}R_{j}^{2}-\frac{d(d+1)}{4}\right]$$

Here, d is the number of algorithms under comparison (here *d* = 4). N is the number of datasets, (*N* = 20, number of subjects). *R_j_* is the rank of the *j*th classifier algorithm based on % Acc as shown in Table 7. The $\chi_{F}^{2}$ value has been computed to be 15, which is greater than $\chi_{F}^{2}{\;}_{\left(3,\;0.01\right)}=11.34$, i.e., the $\chi_{F}^{2}$ value for 3 degrees of freedom with a confidence interval of 99% percent. Thus, with 99% confidence we can say that these classifiers do not perform equally for the present scenario. So, the classifiers’ performance is ranked according to *R_j_*, as seen in Table 7, where our classifier model (LWL-ridge) tops the rank list. This test statistically validates our choice of classifier model compared to these standard classifiers. It is also evident from Table 6 that our classifier model has outperformed other classifiers with respect to every metric.

### 4.6. Evaluation of System Performance on an External Cohort

To infer the integrity and robustness of the devised algorithm, we collected data from an independent external cohort who never took part in the previous experiments. Twenty new volunteers (7 females and 13 males, 10 smokers and 10 nonsmokers) with no history of major pulmonary artifacts (inferred from TCS annual health check-up program) and a moderately active lifestyle from outside our lab took part in this second phase (Cohort-2). The second part of Table 1, i.e., “Cohort-2”, reveals the demographics of the subjects who participated in this phase.

The external cohort was chosen so that the average age was slightly less than the actual participants in order to look at the smoking effects more clearly when there is most likely no other disease involved; older age groups may have other diseases that may introduce confusing markers. Only one set of data was collected from an individual, and hence a total of 20 instances were fed to the decision-making pipeline. Table 8 lists the performance of the originally devised LWL-ridge regression on the independent external cohort comprising of 20 instances (of 10 smokers and 10 non-smokers) when all of the parameters of LWL-ridge are kept unchanged.

Table 8 shows slight degradation of the performance metrics in comparison to the application of the LWL-ridge model on the original dataset (as shown in the 1st row of Table 3). In this regard, it is to be noted that the external cohort consists of 20 datasets (as opposed to 60 datasets in the actual experimentation). The performance of the LWL-model deteriorated marginally; however, it still yielded an accuracy of more than 80% along with a comparably high TPR and TNR. The F score is higher in this case compared to the original dataset. The standard deviations were reduced in this cohort. These results suggest the efficacy and robustness of our approach even in a dataset with the only major risk factor likely to be smoking.

### 4.7. Comparison with Relevant State-of-the-Art

To the best of the authors’ knowledge, healthy and unhealthy lung conditions, based on smokers and non-smokers, have not yet been characterized by analyzing tidal breathing patterns. However, researchers have computed tidal breathing attributes and have tried to characterize respiratory ailments, such as COPD, asthma, pulmonary fibrosis, and/or other airway obstructive conditions [15,16,17]. Smoker and non-smoker detection has also been attempted through various techniques [48,61,62,63,64], but has not been attempted through tidal breathing analysis. In this subsection, we present a comparative analysis of our system with respect to relevant state-of-the-art works related to two broad categories: (a) lung condition monitoring, and (b) detection of smokers.

In Table 9, we have listed the contextual work which takes into account physiological parameters related to tidal breathing to study compromised adult lungs. Some of the parameters used in these studies are listed below.

TTOT = Total time of one complete breathing cycle = TI +TE
TPTEF/TE = Ratio of TPTEF to TE
TPTIF/TI = Ratio of TPTIF to TI
VT = Tidal volume = TVins + TVexp
VT/TI = Ratio of tidal volume to inspiratory time
IP PEF = Integral of expiratory signal from peak to end.
TP PEF20(80) = Post-peak expiratory flow at time 20%(80%).
VE = Minute ventilation. Volume of air breathed per minute.

All of these studies [15,16,17] have acquired tidal breathing using a spirometer and/or pneumotachogram. They have considered most of the forced breathing parameters, including forced expiratory volume in 1 second (FEV1) and maximal voluntary ventilation (MVV), and have performed statistical comparisons of healthy and unhealthy pulmonary systems. However, this work presents an end-to-end system for acquiring tidal breathing using a novel TBPR device, extracting independent physiological parameters, and automatically classifying an unhealthy or a healthy respiratory system. Post data acquisition, our system analyzes the data and delivers output in less than 10 s. Thus, our system can be suitably employed for instantaneous assessment. We have utilized similar physiological parameters as in [15,16,17] and have been able to recognize persons whose respiratory system is compromised due to smoking with high accuracy and precision.

For the sake of completeness, we compare the performance of our tidal-breathing-based approach with other relevant works restricted to smoker detection only. Table 10 draws a comparison with studies related to smoker and non-smoker classification. These approaches are cumbersome and not appropriate for generating immediate assessment results. Gas chromatography/mass spectrometric approaches [61,63] require the tests to be performed only inside lab conditions. The approach employed in [61] acquired data within 1 hour of smoking, whereas the system presented in this paper does not have such a constraint. The MRI approach [48] also does not allow the subject to be tested outside the lab and yielded an accuracy of a mere 69%. The authors in [62] tested the carbon monoxide in the breath of subjects with an average accuracy of 79.6%. This method also cannot be executed in real-time. The paper [64] presented an approach to the acquisition and analysis of exhaled-breath concentrate through enzyme-linked immuno-sorbent assay. It took 20 min for data acquisition and implemented only a statistical analysis. Compared to all these methods, this paper has presented a complete system that deals with the acquisition of tidal expirograms, computes temporal and volumetric features, and has a cascading pattern recognition stage that offers an automated, fast, highly accurate, precise, and infrastructure-agnostic solution.

## 5. Conclusions

This paper presented a novel approach to discriminate between healthy and compromised pulmonary systems based on adult tidal breathing patterns. For this study, smoking was chosen as the principal modality of lung degradation and the proposed method successfully identified the compromised lungs of smokers. Thus, our technique offers a viable, infrastructure-agnostic, and user-friendly approach to the early pre-screening of adult compromised lungs. In addition, the LWL-ridge classification model is tuned properly and implemented so as to negate the need for feature normalization. This approach thus renders the system suitable for handling subjective variations in FRC. As the data acquisition and analysis are executed in less than 70 s, our system is suitable for instantaneous assessment. Our implemented classification approach is also statistically proven to have superior performance compared to the other standard classification techniques. Our devised LWL-ridge classification model yielded comparable performance while operating on a new, independent, external cohort. This further validates the efficacy and robustness of our novel approach to discriminating between healthy and compromised pulmonary systems. A thorough comparison with the relevant state-of-the-art work also reveals the novelty of our systematic design and implementation. The proposed approach is capable of classifying healthy (non-smokers) and unhealthy (smokers) respiratory systems with a high degree of accuracy and precision. This makes our approach suitable for implementation in real-life scenarios to serve as a first-order screening tool for identifying compromised pulmonary systems.

In the future, we plan to work with patients suffering from various lung conditions, such as COPD, asthma, and pneumothorax. We will characterize the various tidal breathing parameters of these patients and will develop strategies to classify patients with various obstructive airway diseases in real-time. We will also increase our participant count and will consider more demographic variances to cater for more diverse data.

## Figures and Tables

**Table 1:** Demographics of Participants.

Demographic Variable	Cohort 1		Cohort 2	
	Smokers	Non-Smokers	Smokers	Non-Smokers
Number	10	10	10	10
Age	35.3 ± 7.96	34.7 ± 6.32	29 ± 7.08	30 ± 7.07
Gender	8 M/2 F	6 M/4 F	9 M/1 F	4 M/6 F
Smoking Years	15.5 ± 6.86	-	13.4 ± 3.04	-
CPD	16.3 ± 4.74	-	14.6 ± 1.94	-
Lifetime-Usage	13.61 ± 9.63	-	10.06 ± 3.12	-

M/F: male/female; CPD: cigarettes per day; Lifetime Usage: measured in pack-years (=CPD × smoking years/20).

**Table 2:** List of used features.

Feature No.	Features	Description
F1	Inspiratory time (TI)	Mean duration of all the acquired Inspiration phases in seconds
F2	Expiratory time (TE)	Mean duration of all the acquired Expiration phases in seconds
F3	Breathing rate (BR)	Number of breaths per minute
F4	Duty Cycle (DCy)	Mean of the ratios of inspiration time to total breath time of all the acquired breath cycles
F5	Peak Inspiratory Flow (PIF)	Maximum flow rate attained during the inspiratory period.
F6	Peak Expiratory Flow (PEF)	Maximum flow rate attained during the expiratory period.
F7	Time to Peak Inspiratory Flow (TP IF)	Mean time from onset to peak of inspiration of all inspiratory phases.
F8	Time to Peak Expiratory Flow (TP EF)	Mean time from onset to peak of expiration of all expiratory phases.
F9	Inspiratory Tidal volume (TVins)	Mean volume of air inspired of all the acquired inspiration phases
F10	Expiratory Tidal volume (TVexp)	Mean volume of air expired of all the acquired expiration phases
F11	Inspiratory velocity (Velins)	Mean velocity of inspiration from onset to peak of inspiration flow of all the acquired inspiration phases
F12	Expiratory velocity (Velexp)	Mean velocity of expiration from onset to peak of expiration flow of all the acquired expiration phases

**Table 3:** Comparison of different classification models.

Scheme	% Acc	TPR	TNR	F	Kappa	AUC	AUP
LWL + L-R	85.0 (18.07)	0.80 (0.3)	0.90 (0.09)	0.70 (0.36)	0.93 (0.09)	0.92 (0.10)	0.92 (0.11)
LWL + L-O	80.83 (11.57)	0.82 (0.15)	0.80 (0.18)	0.81 (0.11)	0.62 (0.23)	0.85 (0.13)	0.86 (0.13)
L-R	64.00 (13.41)	0.60 (0.22)	0.68 (0.16)	0.61 (0.17)	0.28 (0.27)	0.69 (0.15)	0.73 (0.12)
L-O	63.83 (12.33)	0.58 (0.22)	0.70 (0.16)	0.60 (0.18)	0.28 (0.25)	0.67 (0.14)	0.72 (0.13)

L-O = Ordinary Logistic Regression, L-R = Logistic Regression with Ridge regression, TPR = true positive rate, TNR = true negative rate, AUC = area under the receiver operating characteristic curve, AUP = area under the precision-recall curve. Bold blue fonts denote the highest value for each metric (i.e., column-wise).

**Table 4:** Variation of accuracy with *k*, *R*, and percentage of test data.

Choices of {*k*, *R*}	*T* = 1/*t* of the Total Instances				
	(i.e., 60 × 1/*t*) and *t* ∈ {2, 3, …, 7}				
	1/3	1/4	1/5	1/6	1/7
{10, 10^−5^}	65	73.33	100	50	88.89
{5, 10^−4^}	85	80	100	70	88.89
{5, 10^−3^}	85	80	100	70	88.89

**Table 5:** Measures of the performance of classification.

*R*	% Acc	TPR	TNR	F	Kappa	AUC	AUP
10^−3^	86.17 (10.46)	0.84 (0.17)	0.88 (0.14)	0.85 (0.12)	0.72 (0.21)	0.92 (0.10)	0.93 (0.10)
10^−4^	85.02 (18.07)	0.80 (0.3)	0.90 (0.09)	0.82 (0.24)	0.70 (0.36)	0.93 (0.09)	0.90 (0.13)

Bold blue fonts denote the highest value for each metric (i.e., column-wise).

**Table 6:** Statistical measures for the binary classification test with simulated data using SMOTE.

No. of Instances (Actual + Simulated)	% Acc	TPR	TNR	Kappa	AUC	AUP
60 + 60	94.08 (3.87)	0.96 (0.05)	0.92 (0.07)	0.88 (0.08)	0.97 (0.04)	0.96 (0.06)
60 + 120	93.22 (3.37)	0.97 (0.04)	0.90 (0.07)	0.86 (0.07)	0.96 (0.03)	0.95 (0.05)
60 + 180	95.12 (3.38)	0.97 (0.03)	0.94 (0.05)	0.90 (0.07)	0.98 (0.02)	0.98 (0.03)
60 + 240	95.87 (3.02)	0.98 (0.02)	0.94 (0.05)	0.92 (0.06)	0.99 (0.01)	0.99 (0.02)
60 + 300	95.44 (2.65)	0.95 (0.03)	0.95 (0.03)	0.91 (0.05)	0.98 (0.02)	0.98 (0.02)
60 + 360	97.64 (1.64)	0.98 (0.02)	0.97 (0.02)	0.95 (0.03)	1.00 (0.01)	1.00 (0.01)
60 + 420	96.04 (1.91)	0.97 (0.03)	0.96 (0.03)	0.92 (0.04)	0.99 (0.01)	0.98 (0.02)
60 + 480	96.56 (1.80)	0.97 (0.03)	0.96 (0.03)	0.93 (0.04)	0.99 (0.01)	0.99 (0.01)
60 + 540	96.40 (1.48)	0.98 (0.02)	0.95 (0.02)	0.93 (0.03)	0.99 (0.01)	0.98 (0.02)

Bold blue fonts denote the highest value for each metric (i.e., column-wise).

**Table 7:** Comparison with standard classifiers and Friedman Test ranks.

Method	% Acc	TPR	TNR	F	Kappa	AUC	AUP	Rank (Rj)
SVM-RBF	51.67	0.67	0.37	0.55	0.03	0.52	0.51	4
RF	78.33	0.77	0.30	0.76	0.57	0.77	0.79	2.5
kNN	78.33	0.67	0.90	0.72	0.57	0.78	0.76	2.5
LWL-ridge	**86.67**	**0.83**	**0.90**	**0.85**	**0.73**	**0.94**	**0.92**	**1**

Bold blue fonts denote the best value for each metric (i.e., column-wise). SVM-RBF = support vector machine with radial basis function kernel, RF = random forest, kNN = *k*-nearest neighbor, LWL-ridge = locally weighted learning with ridge regression.

**Table 8:** Evaluation of originally devised LWL-ridge classifier on external cohort.

	% Acc	TPR	TNR	F	Kappa	AUC	AUP
**LWL-ridge**	81.33 (8.29)	0.79 (0.25)	0.84 (0.17)	0.79 (0.13)	0.63 (0.17)	0.81 (0.17)	0.85 (0.13)

The values in parentheses are the standard deviations.

**Table 9:** Comparison with the works related to monitoring adult lungs by tidal breathing analysis (TBA).

Year of Study (No. of Subjects)	Tidal Breathing Parameters Utilized	Remarks
Ours (10NS (healthy), 10S unhealthy)	12: TI, TE, BR, DCy, PIF, TPIF, PEF, TPEF, TVins, TVexp, Velins, Velexp	Complete automated system to intelligently recognize smokers from healthy individuals directly from tidal breathing features.
2014 [15] (24 adults with COPD, 13 healthy adults)	TI, TE, BR, DCy, PIF, TPIF, PEF, TPEF, TTOT, TPTEF/TE, TPTIF/TI, VE, VT, IP PEF, TP PEF, TPPEF20, TPPEF80,	Structural analysis of tidal expirograms was carried out to quantify COPD.
2010 [16] (17 adults with COPD, 12 healthy adults)	PEF, VT, VE and several others related to forced breathing	Breath-by-breath structural analysis of expiratory signal during incremental exercise in COPD patients.
2004 [17] (46 juveniles with CF, 25 adults with CF, 21 adults with COPD, 35 healthy adults)	TE, BR, PIF, TPTEF, TVexp, TPTIF, TTOT, TPTEF /TE, IPPEF, TPPEF20, TPPEF80, TPPEF20, TPPEF80,	Inter-relationships between body size, age, and tidal breathing profile in obstructive airway disease was established using multiple linear regression.

COPD: chronic obstructive pulmonary disease. TTOT = Total time of one complete breathing cycle = TI +TE, TPTEF/TE = Ratio of TPTEF to TE, TPTIF/TI = Ratio of TPTIF to TI, VT = Tidal volume = TVins + TVexp, VT/TI = Ratio of tidal volume to inspiratory time, IP PEF = Integral of expiratory signal from peak to end, TP PEF20(80) = Post-peak expiratory flow at time 20%(80%), VE = Minute ventilation.

**Table 10:** Relative comparison with studies aimed towards the detection of smokers.

Year of Study (No. of Subjects)	Modality of Study	Breathing Gesture	Remarks
Ours (10S, 10NS)	Physiological parameters extraction and binary classification	Tidal breathing for 1 min through a hollow, both-sides-open pipe	Classification accuracy 86.17%.
2017 [61] (11S, 7NS)	Forensic analysis via Gas chromatography (GC)/Mass Spectrometry (MC) of breathing signal	Prolonged breaths	12 compounds were determined to be statistically significant between groups. Nicotine was found to be the most significant discriminant. Smokers were detected with an accuracy of 72%, while non-smokers were detected with 100% accuracy. GC/MC analysis took 21 min.
2016 [62] (11S, 9NS)	Environmental carbon monoxide (CO) gas sensor paired with smart Phone	Forceful breaths with 15 secs of breath-hold between inhale and exhale	Twelve statistical features along with several ensemble techniques were used. Average classification accuracy of 79.6%.
2015 [48] (60S, 60NS)	Magnetic Resonance Imaging (MRI) of subjects.	NA	Maximum accuracy obtained was 69.6% with 139 highest-ranked features, SVM-RFE, and 10-fold CV.
2011 [63] (11S, 11NS)	Analysis of breath odor using electronic Nose and GC/MC	One single exhaled breath was collected in a sampling bag.	Principle component analysis (PCA) and Linear discriminant function analysis (LDA) yields 100% accuracy. Forensic analysis of each breath sample took around 35 min.
2004 [64] (11S, 9NS)	Forensic analysis of Exhaled-Breath Condensate (EBC)	Tidal breathing for 20 min	The concentrations of total protein and nitrite and neutrophil chemotactic activity were significantly higher in the EBC of smokers. Only statistical analysis.
